# Supplementary material for: Cholecystectomy Significantly Alters Gut Microbiota Homeostasis and Metabolic Profiles: A Cross-Sectional Study
Source: Nutrients. 2023 Oct 17;15(20):4399. doi: 10.3390/nu15204399 (PMC10609985; doi:10.3390/nu15204399)
Supplement: Supplementary file 1 [file nutrients-15-04399-s001.zip › nutrients-2643525-supplementary.pdf]

1. Materials and Methods

1.1 **Dietary assessment and questionnaires processing**

Dietary intake assessment included whether the food was consumed, consumption frequency (times of per day/week/month/year) and the average amount of food consumption at each time. The 149 food items in the FFQ were classifed into 18 predefned food groups based on similarities in nutrient profle and culinary usage.

1.2 Untargeted Metabolomics

1.2.1 Metabolite sample preparation

Briefly, fecal samples (approximately 50mg) were weighed, 200 microliters of water were added for homogenization, and 800 microliters of pre-cooled (-20℃) solution (methanol: acetonitrile = (1:1, v/v)) were added for protein precipitation, and vortex for 30s. They were then treated with ultrasound in an ice bath (4℃) for 30 minutes. The samples were incubated at -20℃ for 1 hour for secondary precipitation to remove proteins. The obtained supernatant was transferred to a new microfuge tube by centrifugation at a high speed of 15,000rpm for 15 min at 4℃. Samples were concentrated using a SpeedVac system (Thermo Fisher Scientific, MA, USA) and vacuum dried. Before analysis, dried samples were reconstituted in 50% acetonitrile (v/v) and transferred to a glass autosampler vial. For quality control purpose, a QC sample was prepared by pooling 10 uL of every sample.

1.2.2 HPLC-MS analysis parameters

The metabolite samples were analyzed with an UItiMate 3000 HPLC system (Thermo Fisher Scientific, MA, USA) coupled to a high-resolution Q Exactive mass spectrometer (Thermo Fisher Scientific, MA, USA). Chromatographic separation was achieved on Waters ACQUITY UPLC BEH Amide (1.7μm , 2.1×100 mm) column. The flow rate was 0.3mL /min. Mobile phase: Phase A was an aqueous solution containing 25 mM CH_3COONH_4 (ammonium acetate)+25 mM NH_4OH (ammonium hydroxide), and phase B was acetonitrile. The gradient elution procedure is: 0 ~ 1 min, 95 % B; 1 ~ 14 min, 95 %~ 65 % B; At 14 ~ 16 min, 65%~ 40% B; 16 ~ 18 min, 40% B; 18-18.1 min, 40%-95 % B; 18.1 ~ 23 min, 95 % B. The sample size was 2 μL. The sample was kept at 15℃ in the automatic sampler and the column temperature was 40℃.

ESI source parameters were set as follows: spray voltage, 3.5 KV in positive or negative modes,

respectively; sheath gas, 35 arb; aux gas, 15 arb; aux gas heater temperature, 320 °C; capillary

temperature, 320 °C. The Q Exactive was run with polarity switching in full scan ddMS2 mode for all samples and QC samples to acquire MS/MS spectra. The full scan parameters were set as: resolution, 70,000; AGC target, 1e6; maximum injection time, 100 ms; scan range, 70–1050 m/z. The ddMS2 scan parameters were set as: resolution, 17,500; AGC target, 1e5; maximum injection time, 50 ms; scan range, 70–1050 m/z; top N setting, 10; isolation width, 1.5 m/z; collision energy mode, stepped; collision energy type, normalized; normalized collision energy (NCE) at 20, 40, 60 eV; dynamic exclusion duration was set as 7 s for excluding after 1 time.

1.2.3 Untargeted metabolic data analysis

Compound discoverers used to analyze metabolomic data 3.31 [1]. Multivariate analysis was performed in SIMCA 14.1 software (Umetrics, Umea, Sweden). Partial least squares discriminant analysis (PLS-DA), unsupervised principal component analysis (PCA), and orthogonal partial least squares discriminant analysis (OPLS-DA) were constructed to identify discriminatory features in relevant comparisons. The number of permutation tests was set to 200. Metabolic features with variable importance in projection (VIP, VIP > 1, and $p < 0.05$) were reserved as potential differential biomarkers.

1.3 Determination of fecal SCFAs contents

Gas Chromatography-Mass Spectrometry (GC-MS) was used to measure the SCFA content. (GCMS-QP2010 Ultra system, Shimadzu Corporation, Japan). The GC-MS temperature program was as follows: starting temperature 100 °C, increased to 140 °C at 7.5 °C min−1, then increased to 200 °C at 60 °C min−1. The concentration of six fatty acids was calculated by the external standard method.

1.4 Determination of BA levels in the fecal

UPLC-Q Exactive system (UPLC: UltiMate 3000); Column: ACQUITY UPLC® HSS T3 (1.8 µm, 2.1 × 100 mm) was used for quantitative analysis of BAs. An aqueous solu-tion of 1 mM ammonium acetate (phase A) and a methanolic solution of 1 mM ammo-nium acetate (phase B) were used for elution

Table S1. The elution gradien.

Time (min)	A	B
0	80%	20%
0-6	80-40%	20-60%
6-25	0%	100%
25-26	0%	100%
26-28	0-50%	100-50%
28-30	50-80%	50-20%
30-32	80%	20%

Flow rate of 0.3 mL/min, column temperature was maintained at 30°C.

1.5 DNA Extraction, 16S rRNA Gene Amplification

DNA was extracted from the samples according to the Earth Microbiome Project (EMP) standard protocols using the FastDNA Spin Kit (MP Biomedicals Ltd., Santa Ana, CA, USA). Subsequently, the V3-V4 hypervariable region of the 16S rRNA gene was PCR-amplified using the universal bacterial primers(341F/806R) and purified using TIANgel Mini Purification Kit (TIANGEN, Beijing, China).

Results

Table S2. Excluded studies.

Authors	Title	Year	16s region	doi	Exclusion Reason
Kang Z et al.	Proteobacteria acts as a pathogenic risk-factor for chronic abdominal pain and diarrhea in post-cholecystectomy syndrome patients: a gut microbiome metabolomics study.	2019	V4-V5	10.12659/MSM.915984	Data not available
Xu Y et al.	Disordered Gut Microbiota Correlates With Altered Fecal Bile Acid Metabolism and Post-cholecystectomy Diarrhea.	2022	V3-V4	10.3389/fmicb.2022.800604	Data not available
Yoon W J et al.	The impact of cholecystectomy on the gut microbiota: a case-control study	2019	V3-V4	10.3390/jcm8010079	Data not available
Wang W et al.	Cholecystectomy damages aging-associated intestinal microbiota construction	2018	V3-V4	10.3389/fmicb.2018.01402	Data not grouped
Georgescu D et al.	Dyspepsia and gut microbiota in female patients with postcholecystectomy syndrome	2022	/	10.2147/IJWH.S342882	Data not available
Frost F et al.	Carrying asymptomatic gallstones is not associated with changes in intestinal microbiota composition and diversity but cholecystectomy with significant dysbiosis	2021	V1-V2	10.1038/s41598-021-86247-6	Data not available
Park S et al.	Effects of Bile Acid Modulation by Dietary Fat, Cholecystectomy, and Bile Acid Sequestrant on Energy, Glucose, and Lipid Metabolism and Gut Microbiota in Mice	2022	/	10.3390/ijms23115935	No human samples
Wang Q et al.	Dysbiosis of gut microbiota after cholecystectomy is associated with non-alcoholic fatty liver disease in mice	2021	V4-V5	10.1002/2211-5463.13243	No human samples
Grigor’eva I et al.	Gut Microbiome in a Russian Cohort of Pre- and Post-Cholecystectomy Female Patients	2021	V3-V4	10.3390/jpm11040294	Research methods are not suitable

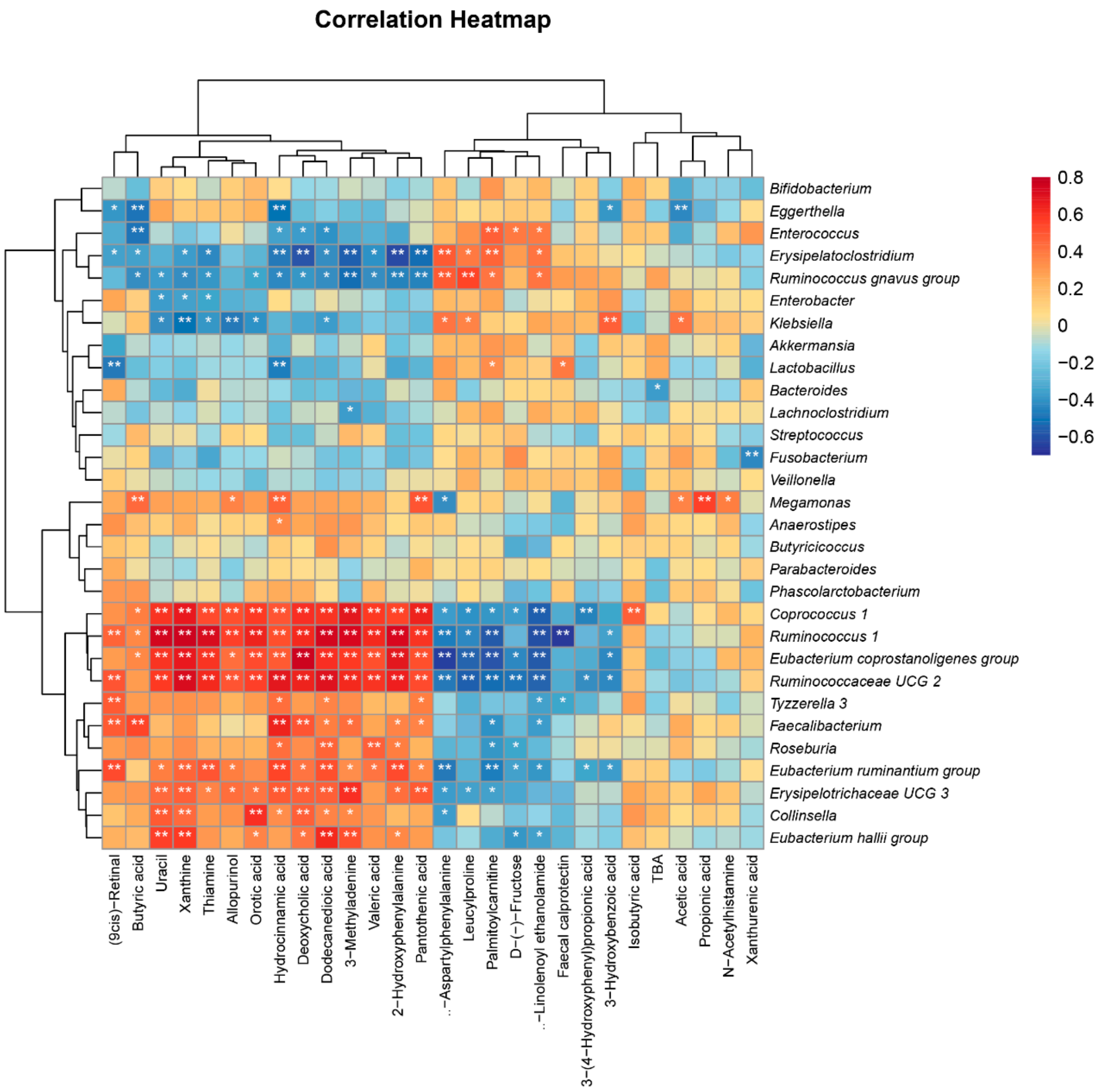

Figure S1 Alterations in fecal metabolic profiles after cholecystectomy as influenced by gut microbiota. Red denotes a positive correlation; blue denotes a negative correlation. The color intensity is proportional to the strength of the Spearman's correlation. *$P \le 0.05$, **$P \le 0.01$, ***$P \le 0.001$.

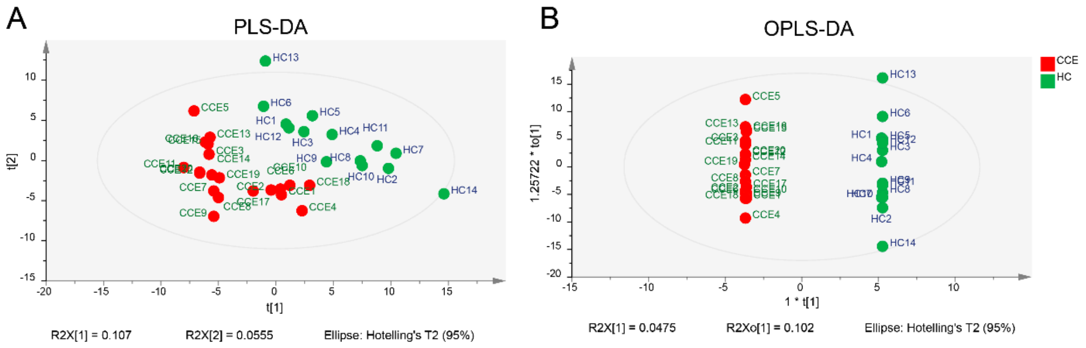

Figure S2 Metabolomics analysis of fecal samples in CCE and HC groups (A). Partial least squares discriminant analysis (PLS-DA). (B) Orthogonal partial least squares discriminant analysis (OPLS–DA).

1. Zhu, G.; Guo, M.; Zhao, J.; Zhang, H.; Wang, G.; Chen, W. Integrative Metabolomic Characterization Reveals the Mediating Effect of Bifidobacterium breve on Amino Acid Metabolism in a Mouse Model of Alzheimer's Disease. *Nutrients* **2022**, *14*, doi:10.3390/nu14040735.
